# Prion potentiation after life-long dormancy in mice devoid of PrP

**DOI:** 10.1093/braincomms/fcab092

**Published:** 2021-04-28

**Authors:** Davy Martin, Fabienne Reine, Laetitia Herzog, Angélique Igel-Egalon, Naima Aron, Christel Michel, Mohammed Moudjou, Guillaume Fichet, Isabelle Quadrio, Armand Perret-Liaudet, Olivier Andréoletti, Human Rezaei, Vincent Béringue

**Affiliations:** 1 Université Paris-Saclay, INRAE, UVSQ, VIM, 78 350 Jouy-en-Josas, France; 2 INRAE, École Nationale Vétérinaire de Toulouse, IHAP, 31 000 Toulouse, France; 3 Neurobiology Laboratory, Biochemistry and Molecular Biology Department, Hôpitaux de Lyon, 69 000 Lyon, France; 4 University of Lyon 1, CNRS UMR5292, INSERM U1028, BioRan, 69 000 Lyon, France

**Keywords:** prion disease, CNS, clearance, misfolded assemblies, transgenic mice

## Abstract

Prions are neurotropic pathogens composed of misfolded assemblies of the host-encoded prion protein PrP^C^ which replicate by recruitment and conversion of further PrP^C^ by an autocatalytic seeding polymerization process. While it has long been shown that mouse-adapted prions cannot replicate and are rapidly cleared in transgenic PrP^0/0^ mice invalidated for PrP^C^, these experiments have not been done with other prions, including from natural resources, and more sensitive methods to detect prion biological activity. Using transgenic mice expressing human PrP to bioassay prion infectivity and RT-QuIC cell-free assay to measure prion seeding activity, we report that prions responsible for the most prevalent form of sporadic Creutzfeldt–Jakob disease in human (MM1-sCJD) can persist indefinitely in the brain of intra-cerebrally inoculated PrP^0/0^ mice. While low levels of seeding activity were measured by RT-QuIC in the brain of the challenged PrP^0/0^ mice, the bio-indicator humanized mice succumbed at a high attack rate, suggesting relatively high levels of persistent infectivity. Remarkably, these humanized mice succumbed with delayed kinetics as compared to MM1-sCJD prions directly inoculated at low doses, including the limiting one. Yet, the disease that did occur in the humanized mice on primary and subsequent back-passage from PrP^0/0^ mice shared the neuropathological and molecular characteristics of MM1-sCJD prions, suggesting no apparent strain evolution during lifelong dormancy in PrP^0/0^ brain. Thus, MM1-sCJD prions can persist for the entire life in PrP^0/0^ brain with potential disease potentiation on retrotransmission to susceptible hosts. These findings highlight the capacity of prions to persist and rejuvenate in non-replicative environments, interrogate on the type of prion assemblies at work and alert on the risk of indefinite prion persistence with PrP-lowering therapeutic strategies.

## Teaser

Abbreviated summary

Martin et al. report lifelong persistence of sporadic Creutzfeldt–Jakob disease prions in the brain of mice lacking the endogenous prion protein gene and disease potentiation—without strain evolution—on retrotransmission to humanized mice. These findings highlight the risk of indefinite prion persistence with therapeutic strategies lowering the prion protein.

## Graphical Abstract

**Graphical Abstract:**
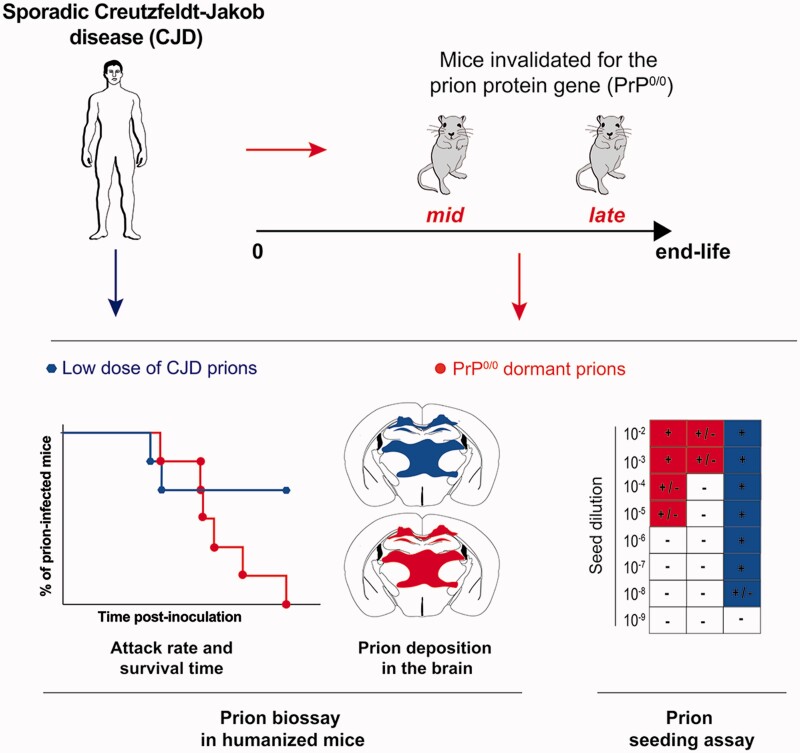

## Introduction

Prions are proteinaceous pathogens that infect the CNS and cause fatal neurodegenerative diseases in human and farmed or wild animals.^1^ Neurons are the primary target cells for prion replication, but supporting glial cells, notably astrocytes can also be infected. Both prion replicative information and pathogenicity (e.g. which CNS regions will be impacted) are encoded in the structure of PrP^Sc^ assemblies, a misfolded conformer of the ubiquitously expressed, host-encoded prion protein PrP^C^.^2^ In infected species, prions self-replicate by a mechanism in which PrP^Sc^ templates PrP^C^ conformational conversion and polymerization. Multiple strains of prions are recognized phenotypically in defined hosts, due to structurally distinct PrP^Sc^ conformers.^3–6^ There is clear evidence that a single strain is composed of structurally heterogeneous PrP^Sc^ assemblies or substrains with markedly distinct, biochemical/biophysical properties and biological activity.^7^ PrP^Sc^ assembly diversification may occur during the pathogenesis and participate to neurotoxicity and adaptation.^8–12^ At the molecular level, PrP^Sc^ assemblies are formed from elementary bricks of PrP.^13^ These sub-elements, termed suPrP are of low-size and exhibit strong resistance to denaturant treatments such as urea.^13^ PrP^Sc^ assemblies are in dynamic equilibrium with suPrP. Such dynamics may contribute to PrP^Sc^ assembly diversification process.^12^

Quantifying prion concentration in a test sample has for long relied on time-consuming bioassays in animals. Prion infectivity titre can be obtained either by end-point titration of the sample in the reporter animals or by using incubation time values as a measure of a titre, once the prion dose–response curve has been established.^14^ Alternatively, and in an accelerated way, cell-free assays estimate prion concentration by measuring prion self-converting activity. In the protein misfolding cyclic amplification assay^15^ and the real-time quaking-induced conversion (RT-QuIC) assay,^16^^,^^17^ the test sample is mixed with a substrate containing PrP^C^ or recombinant, monomeric PrP, respectively, and submitted to cycles of sonication (protein misfolding cyclic amplification) or shaking (RT-QuIC) and quiescent incubation. If the sample contains PrP^Sc^ seeds, PrP^C^ and recombinant PrP will be converted into PrP^Sc^ or into amyloid aggregates, respectively. In the RT-QuIC assay, the presence of amyloid assemblies is followed in real-time by incorporation of thioflavin T, an amyloid-sensitive fluorescent dye. Both tests usually detect sub-infectious doses of prions—thus exhibit greater sensitivities than those of the animal bioassays—and have a wide range of fundamental and applied applications, including prion inactivation studies and diagnostics.^18–20^

Prions can persist in the environment (soil or aqueous) for years.^21–24^ Within tissues and in particular within the brain of challenged animals that are not permissive to prions, there is a limited amount of information on prion fate. Seminal experiments demonstrated that PrP^0/0^ mice, in which the gene encoding PrP^C^ (*Prnp*) has been disrupted, are absolutely resistant to infection by mouse-adapted prions, derived from sheep scrapie^25–27^ or human Creutzfeldt–Jakob disease (CJD).^28^ Measurements of residual prion infectivity in the brain of the PrP^0/0^ challenged animals by bioassay in indicator wild-type mice revealed that these prions were rapidly eliminated within weeks post-challenge.^25^^,^^26^ Resurgent bursts of infectivity were occasionally measured in bio-indicator mice 20–30 weeks after challenge. The authors interpreted these results as the presence of residual infectivity or ‘inadvertent cross-contamination’.^25^^,^^28^^,^^29^ Whether other prions, including from those natural resources would exhibit similar clearance rates in PrP^0/0^ mice remains unknown.

Here, we re-investigate the issue of prion persistence in PrP^0/0^ mouse brain using human prions responsible for the most prevalent form of sporadic Creutzfeldt–Jakob disease (MM1-sCJD sub-type), an animal bioassay using transgenic mice overexpressing human PrP and the highly sensitive RT-QuIC assay. We show that MM1-sCJD prions can persist for the entire life in PrP^0/0^ mouse brain with disease potentiation on retrotransmission to humanized mice.

## Methods

### Prion-infected samples

MM1-sCJD Fr2 sample (frontal cortex extract)^30^^,^^31^ was provided by our collaborators (A.P.L. and I.Q.) within the frame of the French National Neuropathology Network for CJD, based on availability of autopsy-retained frozen brain material and informed consent from the relatives of patients for autopsy and research use, according to French regulations (L.1232–1 to L.1232–3, Code Santé Publique). MM1-sCJD UK1 sample, a WHO reference material (frontal cortex extract),^30^^,^^32^ was provided by the UK National Institute for Biological Standards and Control (CJD Resource Centre, NIBSC, South Mimms, Potters Bar, EN63GG, UK, NHBX0/0001).

### Animal experiments

All the experiments involving animals were carried out in strict accordance with EU directive 2010/63 and were approved by INRAE Local Ethics Committee (Comethea; permit numbers 12–034 and 15–056). PrP^0/0^ mice were the so-called Zurich 1 line.^25^^,^^33^ The human PrP tg650 line has previously been described.^30^ This line is homozygous with about 6-fold overexpression of human PrP^C^ (Met129 allele) in the brain. These mice do not develop any abnormal phenotype or neurological signs with aging and have a normal life span around 2–2.5 years. They do not develop any spontaneous prion disease upon inoculation with uninfected brain material.^30^^,^^34^ Only PrP^0/0^ and tg650 females were used; they were 6–8 weeks old at the time of inoculation. All mice were group housed by 3–5 in polypropylene cages in a standard temperature- and humidity-controlled biosafety laboratory 3 animal facility with a 12-h light-dark rhythm, unlimited access to food and water and enrichment (igloos, wood toys, nests). Cages, food, enrichment and water were sterilized before use.

### Mouse bioassays

To avoid any cross-contamination, a strict protocol was followed, based on the use of disposable equipment and preparation of all inocula in a class II microbiological cabinet. MM1-sCJD Fr2 was prepared at 10% w/v brain homogenate in 5% w/v glucose with a Precellys (Ozyme, Montigny-le-Bretonneux, France). MM1-sCJD UK1 was directly provided at 10% w/v homogenate in 5% glucose.

Two groups of individually identified PrP^0/0^ mice (9 mice per group) were intracerebrally inoculated with 20 µl of UK1 or Fr2 brain homogenate, using a 27-gauge disposable syringe needle inserted into the right parietal lobe. Animals were anaesthetized with 3% isoflurane during the procedure and disposed on a heating pad until they fully recovered. They were monitored daily for general health. They were euthanized at defined time points or at end-life by cervical column disruption. Their brains were carefully collected with separate, disposable tools, homogenized at 20% w/v in 5% glucose and stored at −80°C until further use. For bioassay, 20 µl of the solution were intracerebrally reinoculated at 10% w/v to groups of individually identified tg650 mice (5–7 mice per group). The inoculation procedure was the same as above. Animals were supervised daily for the appearance of neurological signs associated with the development of a prion disease. Animals at terminal stage of disease or at end-life were euthanized by cervical column disruption. Terminal stage criteria for MM1-sCJD strain in tg650 were defined as severe kyphosis, severe ataxia, lethargy, inability to reach food or water and irreversible dorsal decubitus. Brains were collected and homogenized at 20% w/v (for immunoblotting) or directly frozen on dry ice (for histoblotting) before storage at −80°C or fixed by immersion in neutral-buffered 10% formalin (for lesion profiling). The same procedure was followed for sub-passaging in tg650 mice.

For titration of MM1-sCJD or tg650-passaged MM1-sCJD brain infectivity,^30^ groups of individually identified tg650 mice (4–6 mice per group) were inoculated intracerebrally (20 µl) with serial 10-fold dilutions of brain homogenates prepared in 5% w/v glucose solution containing 5% w/v bovine serum albumin, using the same procedure as above. Animals inoculated with the initial dose at 10% were assigned an infectious dose (ID) of 10^−1^. The mice were monitored daily, euthanized at terminal stage or at end-life. Their brains were carefully collected with separate, disposable tools, homogenized at 20% w/v in 5% glucose and stored at −80°C for immunoblot analyses. Infectivity titres (ID_50_; dose that infects half the challenged animals) were calculated by the Spearman−Kärber method.^35^ For comparison with the RT-QuIC assay, the titres were expressed as ID_50_ per ml of 10% (w/v) brain homogenate.

### Western blot

PrP^res^ was extracted from 20% brain homogenates with the Bio-Rad TeSeE detection kit, as previously described.^30^^,^^31^ Briefly, 200 μl aliquots were digested with proteinase K (200 μg/ml final concentration in buffer A) for 10 min at 37°C before precipitation with buffer B and centrifugation at 28 000 × *g* for 5 min. Pellets were resuspended in Laemmli sample buffer, denatured, run on 12% Bis-Tris Criterion gels (Bio-Rad, Marne la Vallée, France), electrotransferred onto nitrocellulose membranes, and probed with 0.1 μg/ml biotinylated anti-PrP monoclonal antibody Sha31 antibody (human PrP epitope at residues 145–152),^36^ followed by streptavidin conjugated to horseradish peroxidase. Immunoreactivity was visualized by chemiluminescence (Pierce ECL, Thermo Scientific, Montigny le Bretonneux, France). The size and relative amounts of PrP^res^ glycoforms were determined using Image Lab software after acquisition of chemiluminescent signals with the Chemidoc digital imager (Bio-Rad, Marne la Vallée, France).

### Histoblots

Brain cryosections were cut at 8–10 μm (NX-70, MM, Lyon, France), transferred onto Superfrost slides and kept at −20°C until use. Histoblot analyses were performed on 2–3 brains per passage, as previously described, using the 3F4 anti-PrP antibody (human PrP epitope at residues 109–112).^37^ Analysis was performed with a digital camera (Coolsnap, Photometrics, Paris, France) mounted on a binocular glass (SZX12, Olympus, Paris, France).

### Vacuolar lesion profiles

Haematoxylin–eosin-stained paraffin-embedded brain tissue sections were used to establish standardized vacuolar lesion profiles in mice, as previously described.^38^^,^^39^ Analyses were performed on 3–5 brains per passage.

### RT-QuIC

RT-QuIC amplifications were performed as previously described.^17^^,^^40^ Briefly, 2 µl of 10% brain homogenates (i.e. 10^−1^ ID) were serially diluted in 20 mM sodium phosphate buffer pH 7.4, 130 mM NaCl, 0.1% SDS and 1× N2 supplement (Thermo Fisher, France). Then, 2 µl of each dilution were loaded in individual wells of a black 96-well optical bottom plate containing 98 µl of 20 mM sodium phosphate buffer pH 7.4, 300 mM NaCl, 10 µM thioflavin T, 1 mM EDTA and 100 µg/ml of purified recombinant human PrP, Met129 allele.^41^ The plate was sealed using Nunc Amplification Tape (Nalgene Nunc International, France), placed in a Xenius XM spectrofluorometer (Safas, Monaco) and incubated for 48–60 h at 47°C. Until the end of the measurements, cycles of 1-min orbital shaking (600 rpm) and 1-min rest were applied, and the fluorescence was recorded every 30 min. Experiments were performed in triplicates or pentaplicates. Each curve was fitted with the following equation:

$$Y=Y\min+\frac{Y\max\;.{\;X}^{h}}{K+\;X^{h}}$$

using MATLAB (R2018b, MathWorks) where *Y* is the fluorescence intensity and *X* the time. The following parameters were then calculated from the fit: fluorescence intensity maximum, slope at the inflexion point and lag time (estimated by extending the tangent at the inflexion point to the initial baseline *Y*min; see Supplementary Fig. 6).

The thresholds used to determine RT-QuIC positivity were obtained from unseeded reactions in which a fluorescence increase was observed (see Supplementary Fig. 6). The means ± SEM values obtained were 28.8 ± 1.5 h for the lag time, 32.5 ± 2.8 for the fluorescence intensity maximum and 3 ± 1 h^−1^ for the slope at the inflexion point. If one of these parameters from a RT-QuIC reaction differed from these thresholds, the RT-QuIC reaction was considered positive.

Seeding activity titre (SD_50_; seeding dose giving thioflavin T positivity in 50% of the replicates) was estimated by the Spearman–Kärber method.^35^ When <100% of the RT-QuIC reactions seeded with the first dilution scored positive or when no dilution scored 100% positive, a trimmed variant of the Spearman–Kärber method was applied.^42^ For comparison with the bioassay, the values were expressed as SD_50_ per ml of 10% (w/v) brain homogenate.

### Statistical analysis

GraphPad Prism 9.0 software (GraphPad, La Jolla, CA, USA) was used to establish the Kaplan–Meier curves plotting the percentage of mice without prion disease against the incubation time. This software was also used to draw the RT-QuIC graphs and vacuolar profiles.

### Data availability

All relevant data are within the manuscript and its supporting information files. Data are fully available without restriction.

## Results

### Lifelong persistence of CJD infectivity in PrP^0/0^ mice

**Figure 1:**
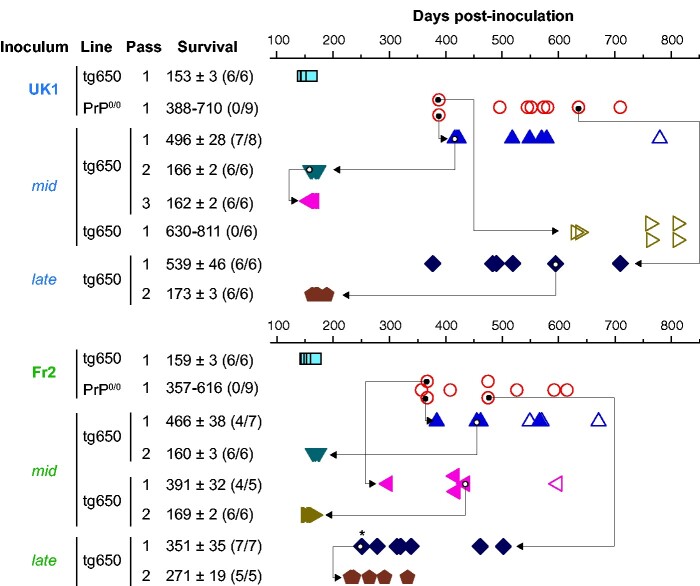
Bioassay of PrP^0/0^-derived MM1-sCJD prions in human PrP mice. Intracerebral inoculation of 2 different cases of MM1-sCJD (UK1, Fr2) to human PrP mice (tg650 line; blue square) and PrP^0/0^ mice (red circle) and back passage of *mid* and *late* PrP^0/0^ brains to human PrP mice (other symbols). Each symbol represents an individual mouse. Closed symbols represent diseased, PrP^res^-positive mice and open symbols represent asymptomatic, PrP^res^-negative mice. The *mid* and *late* mouse brains used for retransmission and further iterative passage in tg650 mice are indicated by the arrow. Survival is expressed as mean ± SEM days; in parenthesis number of diseased, PrP^res^-positive mice/number of inoculated mice. For PrP^0/0^ mice or non-responder groups of tg650 mice, the range of survival time is given. * indicates mouse with lower PrP^res^ content, as further confirmed by a delayed second passage compared to the others.

**Figure 2:**
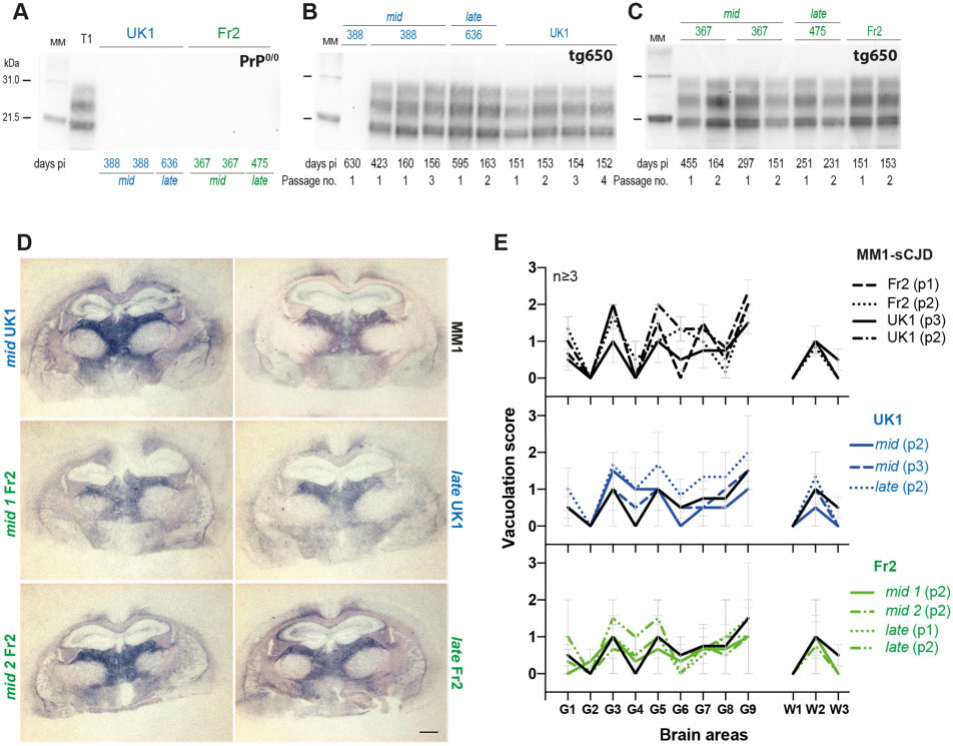
T1 prion phenotype re-emerges from PrP^0/0^-derived MM1-sCJD prions. (**A**) Western blot showing the absence of PrP^res^ in *mid* and *late* brains from PrP^0/0^ mice inoculated with UK1 and Fr2 MM1-sCJD cases and euthanized healthy at the indicated days post-inoculation (pi). T1 PrP^res^ is shown on the left of the gel as positive control. (**B** and **C**) Electrophoretic pattern of PrP^res^ in the brains of tg650 mice euthanized at end life or disease terminal stage (at the indicated days) on primary or serial passage of *mid* and *late* PrP^0/0^ brains from UK1 (**B**) and Fr2 (**C**). As control, the electrophoretic patterns found on direct, serial passaging of UK1 and Fr2 in tg650 mice are shown (right panel of the gels). The passage number (no.) is indicated. Immunoblots were probed with Sha31 anti-PrP monoclonal antibody. MM = molecular mass markers. The original, uncropped gels are shown in Supplementary Fig. 1. (**D**) Histoblot analyses of PrP^res^ neuroanatomical distribution in tg650 mice challenged with *mid* and *late* PrP^0/0^ brains from UK1 and Fr2 as compared to direct inoculation of UK1MM1-sCJD (MM1). Representative histoblots are shown at the level of the hippocampus/thalamus where the neuroanatomical MM1-sCJD signature is the most specific (see Supplementary Fig. 2 for the histoblots in four standard antero-posterior sections). Histoblots were probed with 3F4 anti-PrP monoclonal antibody. Scale bar, 1 mm. (**E**) Standard vacuolar lesion profiles in the brains of tg650 mice inoculated with *mid* and *late* PrP^0/0^ brains from UK1 and Fr2 as compared to direct inoculation of UK1 and Fr2 MM1-sCJD. Analyses were performed after two or three iterative passages (p, as indicated) on three to five brains. The vacuolation intensity was scored as means ± SEM in standard grey (G1–G9) and white (W1–W3) matter areas. G1: Dorsal medulla; G2: Cerebellar cortex; G3: Superior colliculus; G4: Hypothalamus; G5: Medial thalamus; G6: Hippocampus; G7: Septum; G8: Medial cerebral cortex at the level of the thalamus; G9: Medial cerebral cortex at the level of the septum; W1: Cerebellar white matter; W2: White matter of the mesencephalic tegmentum; and W3: Pyramidal tract.

To challenge PrP^0/0^ mice, we used as inocula two unrelated brain homogenates from MM1-sCJD, one from the United Kingdom (UK1, a WHO reference material) and one from France (Fr2). We previously reported that UK1 and Fr2 homogenates were fully pathogenic for human PrP (Met129) tg650 mice, resulting in minimal disease durations of ∼150–160 days (Fig. 1).^30^ We intracerebrally challenged PrP^0/0^ mice (Zurich 1 line)^25^^,^^33^ with high dose of UK1 and Fr2 [20 µl at 10% (w/v)]. The two infections were performed independently. As expected, none of the inoculated PrP^0/0^ mice developed a neurological disease nor accumulated disease-specific, proteinase K-resistant PrP^Sc^ (PrP^res^) (Figs 1 and 2A; Supplementary Fig. 1).

To document the presence of residual infectivity in sCJD-inoculated PrP^0/0^ mice, we intracerebrally inoculated a cohort of bio-indicator tg650 mice with brain material collected from PrP^0/0^ mice euthanized healthy approximately a year post-inoculation, i.e., at mid-life (*mid*) and at end-life (*late*, 450 to 700 days post-inoculation). Whichever the time of collection, tg650 retrotransmission with PrP^0/0^-passaged UK1 and Fr2 resulted in a stereotyped prion disease in a major proportion of mice, as based on the appearance of neurological signs and accumulation of PrP^res^ in the brain (Figs 1 and 2B and C). With *mid* brains, three out of four retrotransmissions were positive, with 57%, 80% and 87% attack rates. With *late* brains, two out of two retrotransmissions were positive, both with 100% attack rate. The collective mean survival times of the positive mice varied between ∼350 and ∼540 days, with a mean ± SEM of the mean of 449 ± 34 days.

The electrophoretic pattern of PrP^res^ purified from the brain of all positive tg650 mice inoculated with *mid* and *late* samples closely resembled that observed in the brain of tg650 mice on direct inoculation of MM1-sCJD, with predominance of monoglycosylated and unglycosylated PrP^res^ and a 21 kDa migration pattern for unglycosylated PrP^res^ (Fig. 2B and C), a stereotyped pattern referred to as T1 PrP^res^.^43^^,^^44^ This strongly suggested that genuine MM1-sCJD prions persisted in PrP^0/0^ brains.

Serial passage of PrP^0/0^-retropassaged prions was performed in tg650 mice to further compare their strain properties with those of the initial and well-characterized MM1-sCJD prions. This was done by standard strain typing method comparing disease duration, PrP^res^ electrophoretic pattern and neuroanatomical distribution of PrP^res^ and of vacuoles. On sub-passaging, the mean survivals decreased. They established for the most advanced set of transmission (UK1) to ∼160 days (Fig. 1), a mean survival time typical of MM1-sCJD prions directly serially passaged in tg650 mice.^30^ Immunoblotting showed that T1 PrP^res^ accumulated in the brain on serial passage (Fig. 2B and C). The neuroanatomical distribution of PrP^res^ is strain-specific.^45^ Direct and PrP^0/0^-intermediate transmission of MM1-sCJD prions to tg650 mice led to a similar distribution pattern of PrP^res^, from the primary retrotransmission onwards, as studied by histoblotting on antero-posterior coronal brain sections (Supplementary Fig. 2). In particular, PrP^res^ from *mid* and *late* brains accumulated specifically in certain thalamic nuclei (Fig. 2D; Supplementary Fig. 2). This thalamic tropism is pathognomonic of MM1-sCJD prions in tg650 mice.^30^^,^^46^^,^^47^ Other brain areas scored consistently PrP^res^-positive such as the cingulate cortex, the cingulum, the septum, the basal forebrain, the colliculi and the pons (Supplementary Fig. 2). In the posterior thalamic nuclei and in the basal forebrain There were variable levels of PrP^res^ deposition amongst the analysed brains, which may be due to different incubation times between the mice (Supplementary Fig. 2).

Strain-specific vacuolar lesion profiles were established by histological examination.^38^^,^^39^ The vacuolation was relatively limited, and there was some variability in the intensity on serial transmission of *mid* and *late* brains, as on direct transmission of MM1-sCJD prions. In both groups, the profiles were relatively similar, with most intense areas of vacuolation in the thalamus, the superior colliculus, the frontal cortex and the mesencephalic tegmentum (Fig. 2E).

Collectively, these data show life-long persistence of MM1-sCJD prions in PrP^0/0^ mouse brain. The PrP^0/0^-remnant seeds seem to retain the strain memory of the parental prions, suggesting no drastic evolution of the strain structural determinant despite a 1–2-year dormancy in PrP^0/0^ brains. These remnant seeds were efficient to induce disease back in tg650 mice with respect to attack rate, suggesting substantial levels of replicating activity.

### PrP^0/0^-dormant prions and low dose of MM1-sCJD prions show discrepant virulence in human PrP mice

**Figure 3:**
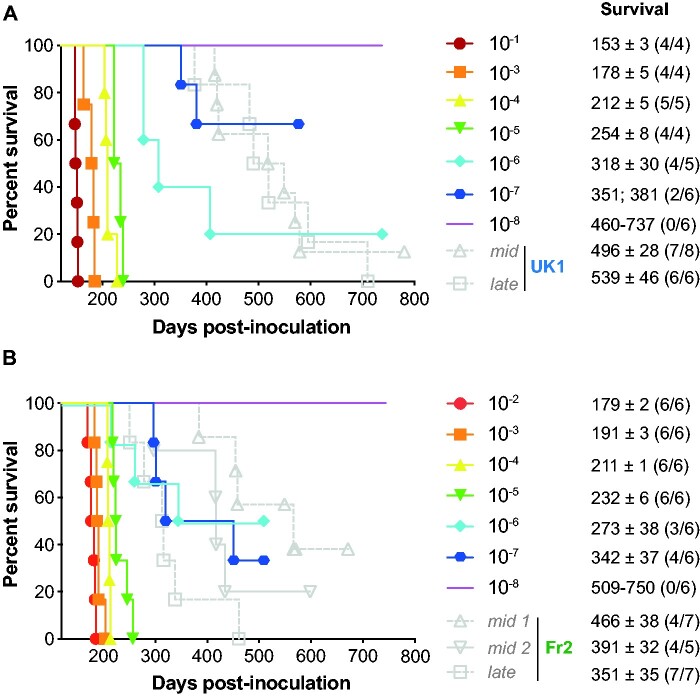
End-point titration of MM1-sCJD prions in human PrP tg650 mice. End-point titration of UK1 brain extract (**A**) or tg650-passaged UK1 (1 passage, (**B**)). Ten-fold dilutions, ranging from 10^−1^ to 10^−8^, as indicated, were intracerebrally inoculated to reporter tg650 mice. The 10^−1^ dilution corresponds to the inoculation of 20 µl 10% (w/v) per mice. Kaplan–Meier curves plot the percentage of mice without prion disease (survival) against the incubation time (days post-inoculation). The different colours and symbols describe the dilutions inoculated. For comparison, grey symbols/dash lines refer to tg650 mice inoculated with *mid* and *late* brains from PrP^0/0^ mice inoculated with UK1 (**A**) and Fr2 (**B**). Survival is expressed as mean ± SEM days; in parenthesis number of diseased, PrP^res^-positive mice/number of inoculated mice. For non-responder groups of tg650 mice, the range of survival time is given.

End-point titration-based bioassay in animals allows determining prion infectivity in tissues.^14^ Such an assay allows correlating the dose of infectious material with the proportion of affected animals and their mean survival times.^48^ To establish such a correlation for *mid* and *late* PrP^0/0^ brains, we titrated UK1 MM1-sCJD CNS material in tg650 mice by limiting dilution. The same material after one passage in tg650 mice (tg650-UK1) was previously titrated and is shown for comparison.^30^ The two Kaplan–Meier curves describing the survival percentage as a function of time and dose are depicted in Fig. 3. Both titrations provided a consistent picture. As expected, the disease incubation period increased and the probability of infection became smaller with dose decrease. Inoculation of material up to the 10^−5^ dilution resulted in 100% lethality. At the 10^−6^ dilution, lethality was below 100%. The dose at the disease limit established at the 10^−7^ dilution. At this dose, 2/6 and 4/6 animals were PrP^res^-positive (UK1: 351; 381 days; tg650-UK1: 342 ± 37 days). For each dose including the limiting one, individual incubation periods were below the limit value of 400 days for all but one mouse that was euthanized at 451 days post-inoculation (Fig. 3). The Spearman–Kärber method allowed calculating the dose that infects half the challenged animals (ID_50_). UK1 and tg650-UK1 had values of 10^8.3^ and 10^8.7^ ID_50_/ml of 10% (w/v) tg650 brain, respectively (Table 1). Fr2 was not titrated by limiting dilution in tg650 mice. However, Fr2 has similar incubation time as UK1 and tg650-UK1 in tg650 mice^30^ and they share similar seeding activity by RT-QuIC free assay (see below; Table 1). We thus considered UK1 and tg650-UK1 titrations as valid for Fr2.

**Table 1:** End-point titration of MM1-sCJD by RT-QuIC and tg650 bioassay

	Bioassay	RT-QuIC
	ID_50_/ml brain ± Std dev^a^	SD_50_/ml brain ± Std dev
UK1	10^8.3 ± 0.6^	10^10.6 ± 0.4^
Fr2	nd	10^10.2 ± 0.4^
tg650-UK1	10^8.7 ± 0.5^	10^11.0 ± 0.2^

Nd = not done.

a As calculated by the Spearman–Kärber method. In ml of 10% (w/v) brain homogenate.

Remarkably, the disease incidence and/or the incubation periods of the tg650 mice that got sick upon inoculation with *mid* and *late* PrP^0/0^ brains were considerably greater than inferred from UK1 and tg650-UK1 titrations (Fig. 3). Disease occurred at 57 to 87% and 100% attack rates with the positive *mid* and *late* brains, respectively. For UK1 *mid* and *late* brains, twelve out of thirteen PrP^res^ positive mice had individual incubation periods over the limit value of 400 days (Fig. 3A). Among them, nine had incubations periods around or over 500 days. For Fr2 *mid* and *late* brains, the situation was more balanced, seven out of the fifteen mice had incubation periods over 400 days. The other eight mice had incubation periods in the range of the 10^−6^/10^−7^ dilution (Fig. 3B).

The collective titration (by the incubation period bioassay) of tissues containing low amounts of MM1-sCJD prions in our laboratory showed individual incubation periods rarely over 400 days post-inoculation (Supplementary Fig. 3). This is consistent with the end-point titrations (Fig. 3) and further suggests that at low or limiting dose, the disease duration of MM1-sCJD prions in tg650 mice is below 400 days. This 400-day limit is consistent with the fact that in all our end-point titrations performed so far (in the homotypic PrP context), the incubation period fold increase between the incubation duration at the lowest and at the limiting dilution is 2.17 ± 0.32.^8^ Applying this value to MM1-sCJD titration (mean incubation duration between 150 and 160 days)^30^^,^^47^ would result in a theoretical incubation duration value at the limiting dose between ∼280 and ∼400 days. This 400-day limit is also consistent with MM1-sCJD end-point titrations with other transgenic mouse lines expressing human PrP.^49^^,^^50^

Thus, the efficient transmission observed with three *mid* and *late* brains coupled with long incubation periods appears discrepant with respect to the virulence of low doses of MM1-sCJD prions in tg650 mice.

### Low seeding activity of PrP^0/0^-dormant prions

**Figure 4:**
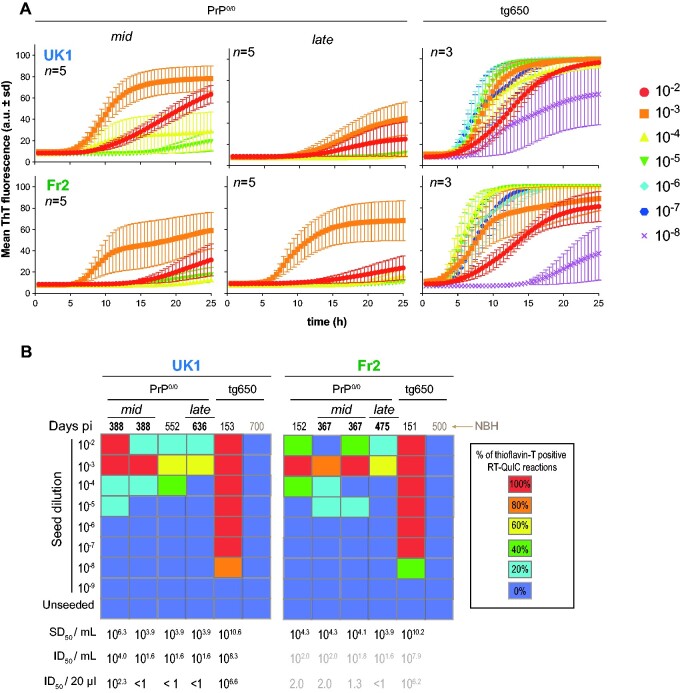
Seeding activity of PrP^0/0^-derived MM1-sCJD prions. (**A**) Representative RT-QuIC reactions obtained by mixing human recombinant PrP with dilutions of brain homogenates from *mid* and *late* PrP^0/0^ mice and tg650 mice challenged with UK1 and Fr2 MM1-sCJD prions. Each sample was serially diluted down to the 10^−8^ dilution. Each trace is the fitted curve plotting the mean ± sd ThT fluorescence intensity over time (recorded every 30 min) from 3 to 5 replicate wells, as indicated. The individual curves are shown as Supplementary Fig. 5. (**B**) Summary of the RT-QuIC experiments. Colour scale in the boxes indicates the % of positive RT-QuIC reactions out of the total number of reactions analysed, as indicated. For each brain tested, the average Spearman–Kärber estimates of the SD_50_/ml of 10% (w/v) brain homogenate are indicated, as well as the extrapolated ID_50_/ml and per inoculated mouse (20 µl), as inferred from Table 1. For Fr2, extrapolation is presented as grey values, as no direct measure of the ID_50_ was available. NBH = Normal brain homogenate.

To provide further quantitative estimates of prion concentration in PrP^0/0^ brains, we examined the remnant MM1-sCJD seeding activity in *mid* and *late* PrP^0/0^ brains relative to MM1-sCJD seeding activity in the brains of clinically sick tg650 mice inoculated with UK1 and Fr2. Two more PrP^0/0^ brains were analysed compared to the bioassay, at 152 days (Fr2) and 552 days (UK1) post-infection. Ten-fold dilutions of PrP^0/0^ or tg650 brains inoculated with UK1 and Fr2 were mixed with human recombinant PrP and submitted to the RT-QuIC assay.^17^^,^^40^ Representative reactions are shown in Fig.4A and the results are summarized in Fig. 4B. All the individual data are shown as Supplementary Fig. 5. There was no increase in thioflavin T fluorescence up to 20–25 hours when human recombinant PrP was mixed with serial dilutions of aged uninfected tg650 brain (Supplementary Fig. 4; Fig. 4B), suggesting no spontaneous conversion during this period. Starting from 10^2^-diluted 10% brain material mixed (1:50 dilution) with the recombinant PrP and thioflavin T containing buffer for the RT-QuIC reaction, brains from terminally sick tg650 mice inoculated with UK1 and Fr2 showed 100% positive replicates down to the 10^−7^ dilution. The limiting dilution was achieved at the 10^−8^ dilution. These end-point titrations allowed calculating the median seeding dose (SD_50_) per millilitre of tg650 mouse brain homogenate by the Spearman–Kärber method. It established at 10^10.6^ and 10^10.2^ SD_50_ per mL of 10% (w/v) tg650 brain for UK1 and Fr2, respectively (Fig. 4B; Table 1). *Mid* and *late* PrP^0/0^ brains exhibited variable, yet measurable and consistently low seeding activity by RT-QuIC. In short, four out of eight PrP^0/0^ brains achieved 100% positive replicates up to the 10^−3^ dilution. At this dilution, the remaining four brains had 60–80% of positive replicates. The limiting dilution was reached for the eight brains tested at the 10^−3^ (two brains), 10^−4^ (three brains) or 10^−5^ dilution (three brains). We calculated the SD_50_ concentrations in PrP^0/0^ brains by the Spearman–Kärber method and a trimmed variant for <100% response. All but one brain had values between 10^4^–10^5^ SD_50_/ml of 10% (w/v) PrP^0/0^ brain. The remaining brain had a 10^6.6^ SD_50_/of 10% (w/v) PrP^0/0^ brain value (Fig. 4B). There was a 4.3–6.7 Log_10_ (UK1) and a 5.9–6.3 Log_10_ (Fr2) decrease in SD_50_ concentrations between PrP^0/0^- and tg650-passaged MM1-sCJD prions.

Correlating the Spearman–Kärber calculations of the SD_50_ and ID_50_ per ml 10% brain (Table 1) allowed us to extrapolate the theoretical ID_50_ in PrP^0/0^ brains for UK1 experiment. Except for one *mid* brain, the values would be below 1 ID_50_ as 20 µl are inoculated in bio-indicator tg650 mouse (Fig. 4B). A similar extrapolation was made for Fr2 (although no titration was done), based on the similar correlation between SD_50_ and ID_50_ found for both UK1 and tg650-UK1 (see above and Table 1). This would provide theoretical ID_50_ values for Fr2 *mid* and *late* brains below 1 up to 2.0 ID_50_ (Fig 4B). Collectively, the RT-QuIC analyses indicate low remnant seeding activities in *mid* and *late* PrP^0/0^ brains. They allow to extrapolate that the amount of infectivity inoculated to bio-indicator tg650 mice would be below <1 or in the range of 1–2 ID_50_ for all but one brain. Such values would not permit disease at the attack rate we observed, except for the second *mid* UK1 brain (Fig. 1). Thus, the efficacy at which *mid* and *late* brains infect tg650 mice appears discrepant relative to their seeding activity as measured by RT-QuIC.

### Kinetics of PrP^0/0^-dormant prions versus MM1-sCJD prions

Beyond quantitating the seed concentrations in a test sample, the RT-QuIC can compare the seeding activity of closely related prion samples, based on the kinetic parameters of recombinant PrP polymerization reaction. We compared here the lag phase, slope at the inflexion point and final fluorescence intensity values (Supplementary Fig. 6A) of *mid* and *late* PrP^0/0^ brains with those of tg650 brains infected with UK1 and Fr2. The lag time tended to last longer (Supplementary Fig. 6B), the slope at the inflexion time (Supplementary Fig. 6C) and the final fluorescence intensity (Supplementary Fig. 6D) tended to be lowered in PrP^0/0^ brains at equivalent dilutions of brain homogenate. Yet, the brain matrix/seeding particles concentration was not the same between PrP^0/0^-passaged versus tg650-passaged MM1-sCJD and might contribute to the small differences observed, due to potential inhibitory effect of the brain matrix on the seeding of recombinant PrP polymerization reactions.^17^ This suggests that the PrP^0/0^ dormancy has not significantly altered/impacted the converting activity of the seeds.

Finally, the SD_50_ concentrations over time in PrP^0/0^ mice allowed us to calculate PrP^Sc^ half-life in the PrP^0/0^ brain. The values established to 25 days for UK1 and 9 days for Fr2 (Supplementary Fig. S7). At the time of intracerebral inoculation, up to 99% of prion infectivity may escape the brain due to the spill over of surplus inoculum.^51^ Applying this reduction factor to the inoculum SD_50_ value at the time of injection would increase the half-life values to 50 and 15 days, respectively.

## Discussion

In this study, we first asked how long prions persist in PrP^0/0^ brain and remain infectious. We found that MM1 prions responsible for the most common form of sporadic CJD in humans could persist in the brain of PrP^0/0^ mice, for their entire life, as shown by bioassay in human PrP transgenic mice and by measuring their seeding activity by RT-QuIC. These data considerably extend the period for which prions were previously found to persist in PrP^0/0^ brains.^25^^,^^28^ Prion resistance to inactivation is strain-dependent^52^ and MM1-sCJD prions may be more difficult to degrade than the laboratory mouse prions used in these studies. The mouse lines used as bio-indicators for back-passage also differed; we used transgenic mice overexpressing human PrP instead of conventional mice (CD-1 or ddY mice).^25^^,^^26^ Whether overexpression confers higher susceptibility (not simply shortened incubation times) remains to be determined.^34^^,^^53^^,^^54^ Other major differences in the protocols could account for the differences observed between these earlier studies and ours, including notably the fact that brain pools were bioassayed whereas we tested individual PrP^0/0^ brains (no dilution effect, one brain was negative here) and that these brain pools were heated for 20 min at 80°C, thus likely reducing the total amount of infectivity.

Measuring by RT-QuIC prion seeding activity over the mouse lifespan provided a unique opportunity to estimate PrP^Sc^ half-life in PrP^0/0^ brains.^55^ It ranges from 9 to 25 days (15–50 days if inoculum escape from the brain is taken into account), depending on the MM1-sCJD brain analysed. These values are much higher than the 1.5–3 days found upon PrP^C^ expression stoppage in infected cells or mouse brain^56^^,^^57^ and suggest that total prion clearance in PrP^0/0^ brain may be difficult.

Pathogen clearance from neurons is usually non-cytolytic and requires immunologically specific processes.^58^ As prions are formed from abnormal conformations of the host-encoded prion protein, they are immunologically self-tolerated after CNS or extraneural infection of PrP^C^-expressing individuals. To our knowledge, there are no reports of an antibody response mounted in intracerebrally inoculated PrP^0/0^ mice. Early stage of prion infection may stimulate or involve brain innate immune response, through varying molecules and signalling pathways, including Toll-like receptors,^59^ anti-inflammatory cytokines,^60^ interferon-related pathways^61^ and complement.^62^ Such pathways may activate neuron-supporting glial cells such as microglial cells to remove toxic material.^63^^,^^64^ Whether these pathways are activated in prion-inoculated PrP^0/0^ mice and/or are fully functional in the absence of the PrP gene remains unsure.^65^^,^^66^ It may therefore be difficult to eliminate prion from brain territories, particularly in our experimental setup where we used high doses to inoculate the mice.

Second, we investigated in details PrP^0/0^-dormant prions desilencing on back passage to human PrP mice and asked whether their replicative capacities would be altered. We revealed that the probability of reinfection was higher than inferred from their low SD_50_ or ID_50_ concentrations. This efficacy was not palpable in terms of disease incubation periods which were overall aberrantly prolonged but in terms of disease attack rate. Remarkably, the most efficient brains with respect to attack rate were the *late* brains collected at 500–600 days post-inoculation. The underlying processes associated with the ‘rejuvenation’ of PrP^0/0^ dormant prions must accommodate three intricate observations: (i) the PrP^Sc^ assemblies partly escaped total clearance, suggestive of colonization of inaccessible territories and/or existence of conformations allowing ‘absolute’ resistance to catabolism; (ii) on contact with convertible PrP^C^ back into tg650 mice, the dormant assemblies exhibited a replicative advantage compared to freshly diluted counterparts with similar activity, not in terms of fastness to disease, neither in terms of faster converting activity as shown by the RT-QuIC kinetics but in terms of disease incidence. Thus, the dormant assemblies were not the best catalysts but were the best initiators of the disease; (iii) MM1-sCJD strain properties (in tg650 mice) appeared preserved in dormant prions. In other words, these observations imply that the dormant assemblies cannot be considered as hidden, diluted material stored in inaccessible reservoir(s) that simply re-enters the conversion process unchanged. Several hypotheses could be formulated. The first one is a strain evolution during dormancy to a restrained degree of magnitude compatible with PrP^0/0^-derived prions retaining the parental prion strain phenotype in tg650 mice. A second one is the removal (or adsorption as with soil-bound prions?),^23^ during dormancy, of molecules (e.g. carbohydrates, lipids)^67–69^ serving as structural backbone to maintain PrP^Sc^ infectious/virulence properties. Local conformational change in PrP^Sc^ due to removal/addition of molecules may indeed change the pattern of infection.^70^ The third one which is linked to recent compelling evidence that prion assemblies are not a continuum of assemblies of different size with the same core structure.^7^^,^^8^^,^^12^^,^^13^^,^^71^^,^^72^ Synergies between these sub-assemblies are key to prion replication, diversification, and adaptation. A simple urea-induced disassembling process^7^^,^^13^ or dilution process^8^ can alter certain sub-assemblies with respect to their conformation, impacting directly prion biological activity.^7^ Similar phenomenon could occur during life-long dormancy in PrP^0/0^ brain with persistence in the brain of only certain sub-assemblies and elimination of other, that would overall impact the disease pathogenesis.

This last hypothesis would be consistent with our RT-QuIC experiments. In routine use, the RT-QuIC assay exhibits high analytical sensitivity and strong correlation with infectivity bioassay in measuring prion concentration.^17^ Here, the RT-QuIC was able to detect PrP^0/0^-dormant prions but the measured SD_50_ values underestimated their *bona fide* infectiousness. The RT-QuIC assay generates recombinant, thioflavin T positive, PrP amyloid polymorphs that are off-pathway to prion infectivity, at least with wild-type PrP sequence.^73^^,^^74^ PrP^0/0^ life-long dormancy may have specifically preserved the PrP^Sc^ assemblies responsible for prion infectivity and destroyed part of the PrP^Sc^ assemblies responsible for RT-QuIC seeding activity, resulting in aberrantly low SD_50_ values.

Multiple lines of evidence support the view that other neurodegenerative diseases such as Alzheimer’s, Parkinson’s and Huntington’s diseases involve similar mechanisms of misfolding and aggregation of host-encoded polypeptides through a seeded protein polymerization process.^75–79^ The proteopathic seeds involved in the propagation of these diseases can also persist lifelong.^80^^,^^81^ The extreme longevity of prion and prion-like seeds strongly advocate for stringent measures to mitigate accidental or iatrogenic transmission of these diseases by contaminated non-disposable surgical instruments or biologics. From a therapeutic standpoint, the question of whether prions or prion-like proteins can be totally eliminated from the brain is crucial. In prion diseases but also in other neurodegenerative diseases,^82^ therapeutic strategies aimed at lowering/dosing the production of the disease-causing protein are emerging, using chemical compound targeting the unfolded protein response,^83^ anti-PrP antibodies^84^^,^^85^ or antisense oligonucleotides.^86^^,^^87^ Our experiments would suggest that the brains of the treated individuals may remain potentially contagious and that replication would restart once the treatment is lifted. Early intervention before infectivity peaked in the brain and therefore our capacity to diagnose the disease as early as possible to avoid accumulation of too high levels of infectivity is key to the success of these highly promising therapies. Alternatively, multiple treatments/interventions using the above-mentioned strategies and pharmacotherapies for promoting intra-cerebral clearance^88^ may turn out to be the most viable approach to ensure total prion clearance.

## Supplementary material

Supplementary material is available at *Brain Communications* online.

## Supplementary Material

fcab092_Supplementary_Data

## Abbreviations

CJD =: Creutzfeldt–Jakob disease
ID =: infectious dose
PrP^C^ =: cellular form of the prion protein
PrP^Sc^ =: pathological form of the prion protein
PrP^0/0^ =: PrP knock-out
RT-QuIC =: real-time quaking-induced conversion
sCJD =: sporadic Creutzfeldt–Jakob disease
SD =: seeding dose
