# Supplementary material for: Prion potentiation after life-long dormancy in mice devoid of PrP
Source: Brain Commun. 2021 Apr 28;3(2):fcab092. doi: 10.1093/braincomms/fcab092 (PMC8111064; doi:10.1093/braincomms/fcab092)
Supplement: fcab092_Supplementary_Data [file fcab092_supplementary_data.pdf]

Supplementary Data for manuscript:

Prion potentiation after life-long dormancy in mice devoid of PrP

Davy Martin[1], Fabienne Reine[1]*, Laetitia Herzog[1]*, Angélique Igel-Egalon[1], Naima Aron[2], Christel Michel[1†], Mohammed Moudjou[1], Guillaume Fichet[1††], Isabelle Quadrio[3,4], Armand Perret-Liaudet[3,4], Olivier Andréoletti[2], Human Rezaei[1], and Vincent Béringue[1]*

[1]*Université Paris-Saclay, INRAE, UVSQ, VIM, 78 350, Jouy-en-Josas, France*

[2]*INRAE, École Nationale Vétérinaire de Toulouse, IHAP, 31 000, Toulouse, France*

[3]*Neurobiology Laboratory, Biochemistry and Molecular Biology Department, Hôpitaux de Lyon, 69 000 Lyon, France*

[4]*University of Lyon 1, CNRS UMR5292, INSERM U1028, BioRan, 69 000 Lyon, France*

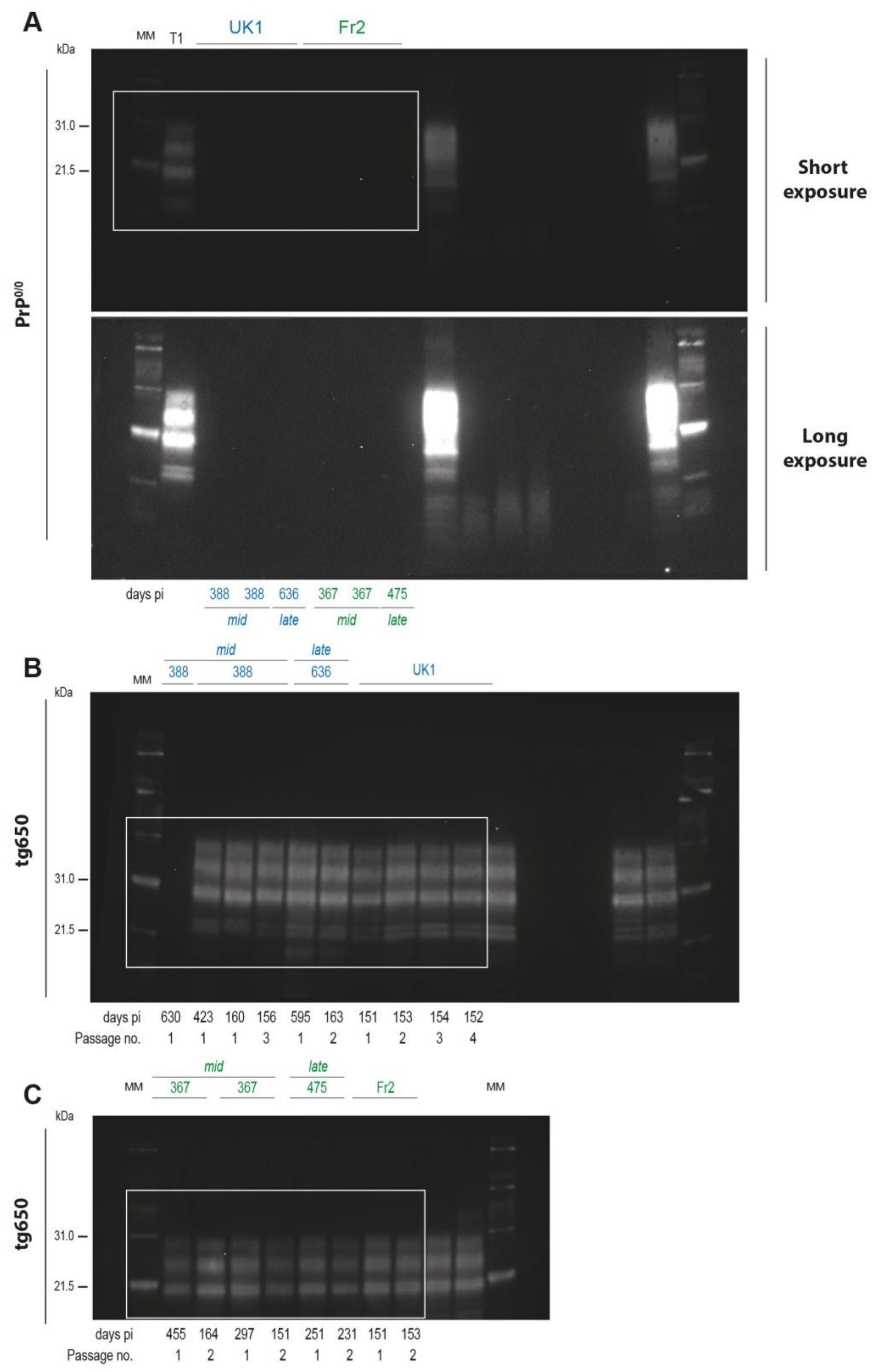

Supplementary Figure 1. Uncropped gels from Figure 2.

PrP^{res} was detected by immunoblot with a digital Imager. Squares indicate the blots that served for illustration in **Fig. 2**.

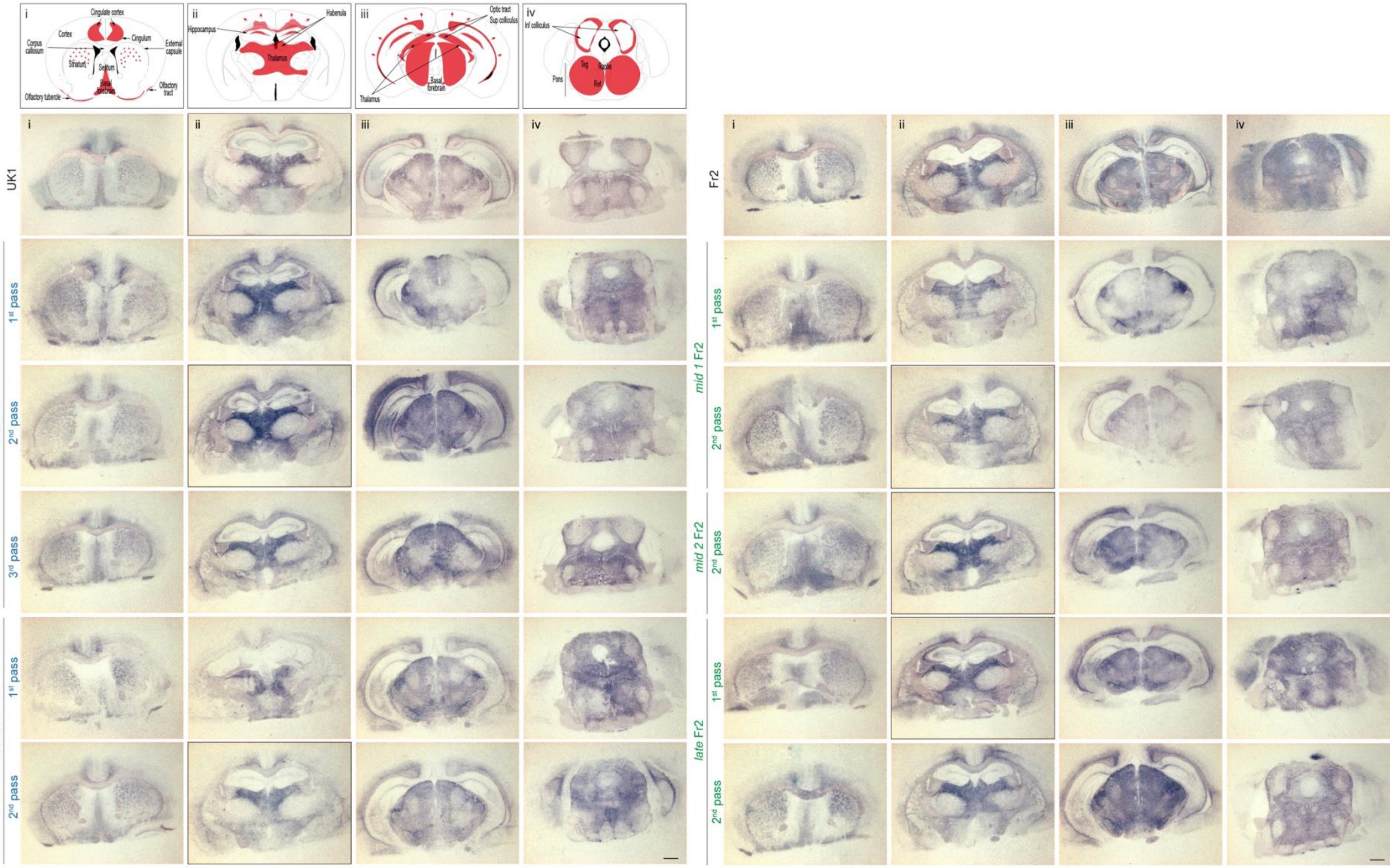
i
Cingulate cortex
Cortex
Cingulum
Corpus callosum
External capsule
Striatum
Septum
Basal forebrain
Olfactory tract
Olfactory tubercle
ii
Habenula
Hippocampus
Thalamus
iii
Optic tract
Sup colliculus
Basal forebrain
Thalamus
iv
Inf colliculus
Teg
Raphe
Pons
Ret
UK1
1st pass
2nd pass
3rd pass
mid UK1
late UK1
Fr2
mid 1 Fr2
mid 2 Fr2
late Fr2

Supplementary Figure 2. Neuroanatomical pattern of PrP^{res} deposition in human PrP tg650 mice inoculated with $PrP^{0/0}$ dormant prions

Tg650 mice were inoculated with UK1 and Fr2 *mid* and *late* $PrP^{0/0}$ brains. Representative histoblots of antero-posterior coronal brain sections are shown at different passages, as indicated, at the level of the septum (i), hippocampus (ii), midbrain (iii), and brainstem (iv). As control, the deposition pattern of UK1[1, 2] and Fr2 in tg650 mice is shown. A schematic representation (adapted from)[3] of the four analyzed zones is shown to highlight common areas of PrP^{res} deposition (in red). Brain ventricles are colored in black.

In section (i), the cingulate cortex, the cingulum, part of the striatum, the septum, the basal forebrain and the olfactory tracts scored PrP^{res}-positive.

In section (ii), the staining was prominent in the thalamus. Several nuclei were positive such as the habenular, paraventricular, lateral posterior, laterodorsal, mediodorsal, central medial and ventromedial nuclei. Others were negative such as the ventrolateral, ventral posteromedial and posterior nuclei. It makes PrP^{res} deposition in the thalamus looking like two symmetrical capital Cs. This is a pathognomonic feature of MM1-sCJD prions in tg650 mice.[1, 4, 5] In this section, the cingulum, the cingulate cortex (as shown by red arrows) and the lacunosum stratum of the hippocampus also scored PrP^{res}-positive.

In section (iii), the cingulum, the external capsule and the surrounding areas (including sometimes the cortex, as shown by red arrows) were PrP^{res}-positive. The optic tract, the superior (Sup) colliculus and the lateral thalamic nuclei scored PrP^{res}-positive. Variable levels of PrP^{res} deposition were seen in the posterior thalamic nuclei and in the basal forebrain.

In section (iv), the inferior (Inf) colliculus and the Pons, notably the tegmental (Teg), reticular (Ret) and Raphe nuclei, scored PrP^{res}-positive.

Histoblots were probed with 3F4 anti-PrP monoclonal antibody. Squares indicate the sections that served for illustration in **Fig. 2**. Scale bar, 1mm.

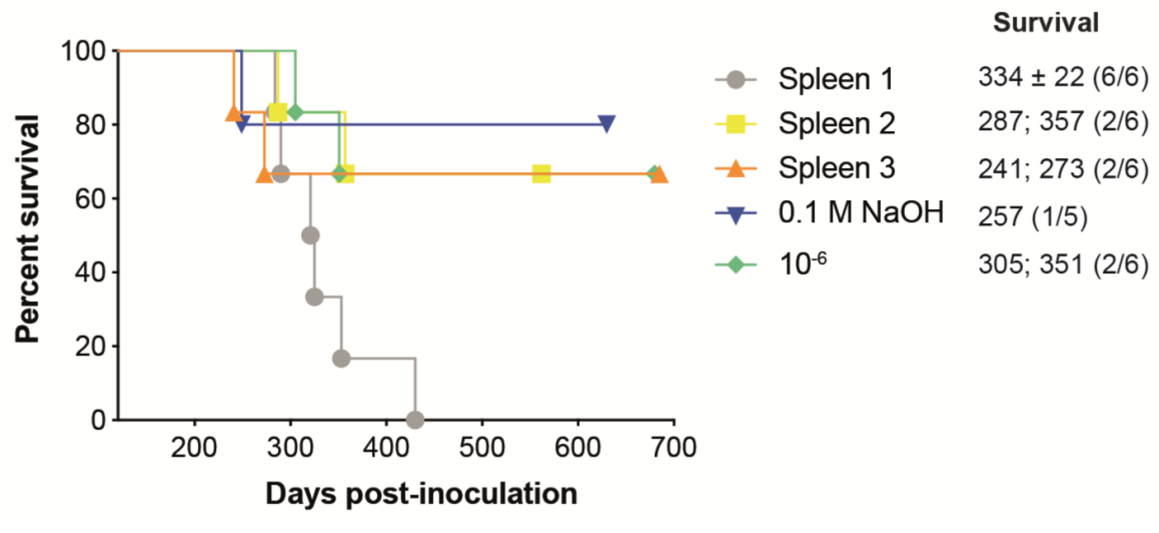

Supplementary Figure 3. Bioassay of extracts containing low dose of MM1-sCJD prions in human PrP tg650 mice

Groups of bio-indicator tg650 mice were intracerebrally inoculated with three different spleens from tg650 mice infected with MM1-sCJD[4], Fr2-brain homogenate treated with 0.1 N NaOH for 1h at room temperature as partial inactivation method, and a 10^{-6} dilution of tg650-UK1. Kaplan-Meier curves plot the percentage of mice without prion disease (survival) against the incubation time (days post-inoculation). The colors and symbols describe the samples inoculated. Survival is expressed as mean ± SEM days; in parenthesis number of diseased, PrP^{res}-positive mice/number of inoculated mice.

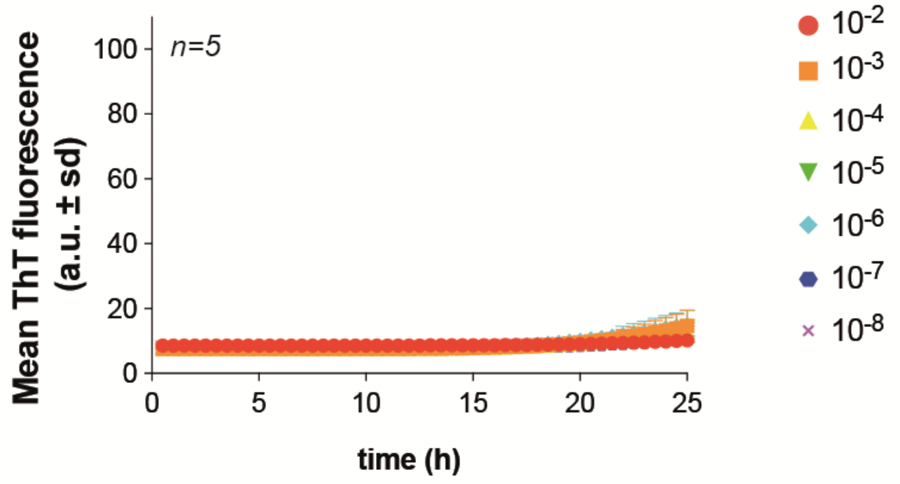

Supplementary Figure 4. No self-polymerization of human recombinant PrP mixed with uninfected tg650 brain homogenate during RT-QuIC reactions

Dilutions of uninfected brain homogenate from aged tg650 mice were mixed with human recombinant PrP and submitted to RT-QuIC reactions. Five replicate wells were used per dilution. The fitted curves plot the mean ± sd ThT fluorescence intensity over time (recorded every 30 min).

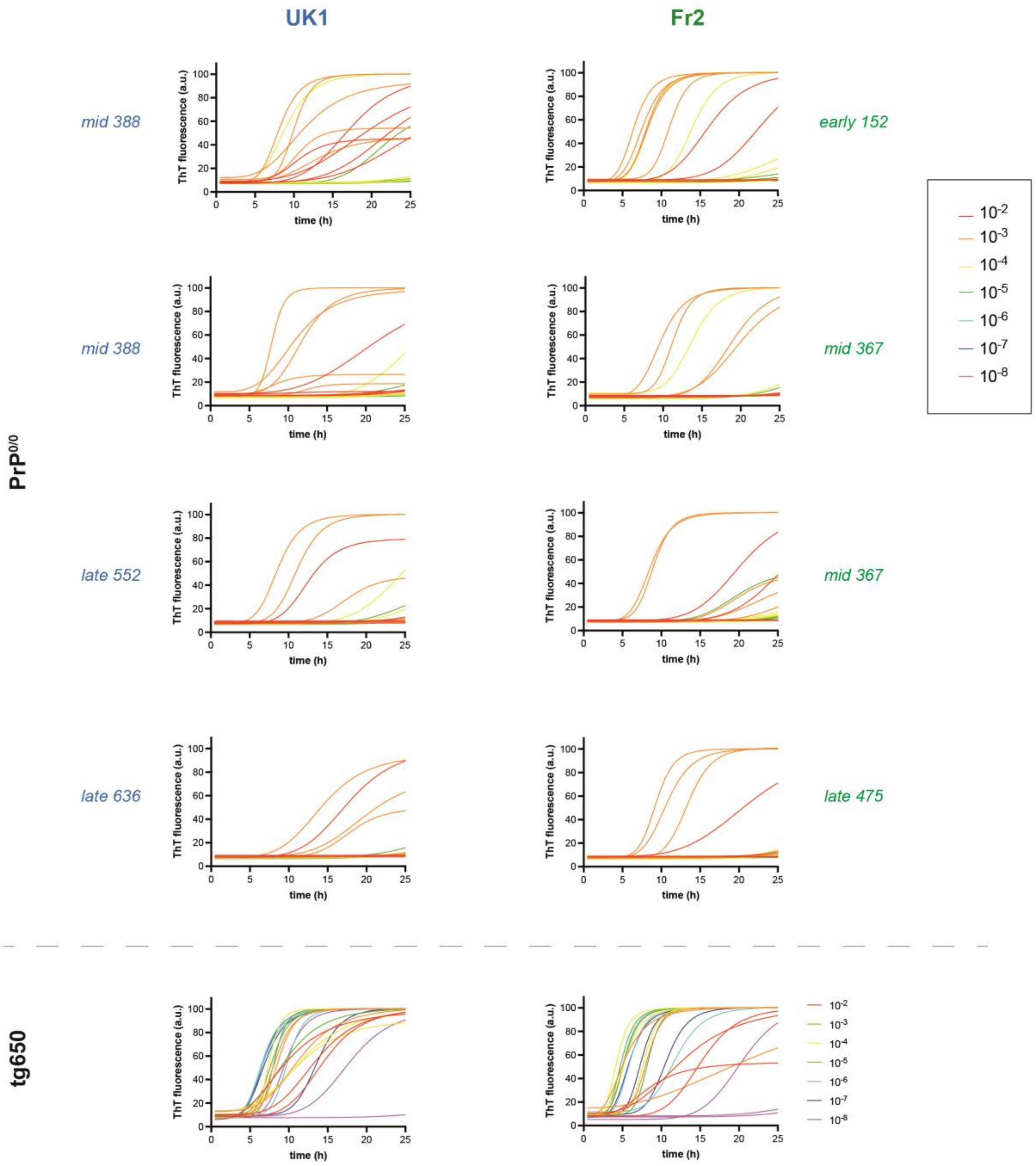

Supplementary Figure 5. Individual RT-QuIC reactions

Individual RT-QuIC reactions obtained by mixing human recombinant PrP with dilutions of brain homogenates from *mid* and *late* $PrP^{0/0}$ mice (n=5) and tg650 mice challenged with UK1 and Fr2 MM1-sCJD prions (n=3). Each sample was serially diluted down to the 10^{-8} dilution. Each trace is the fitted curve plotting ThT fluorescence intensity over time (recorded every 30 min).

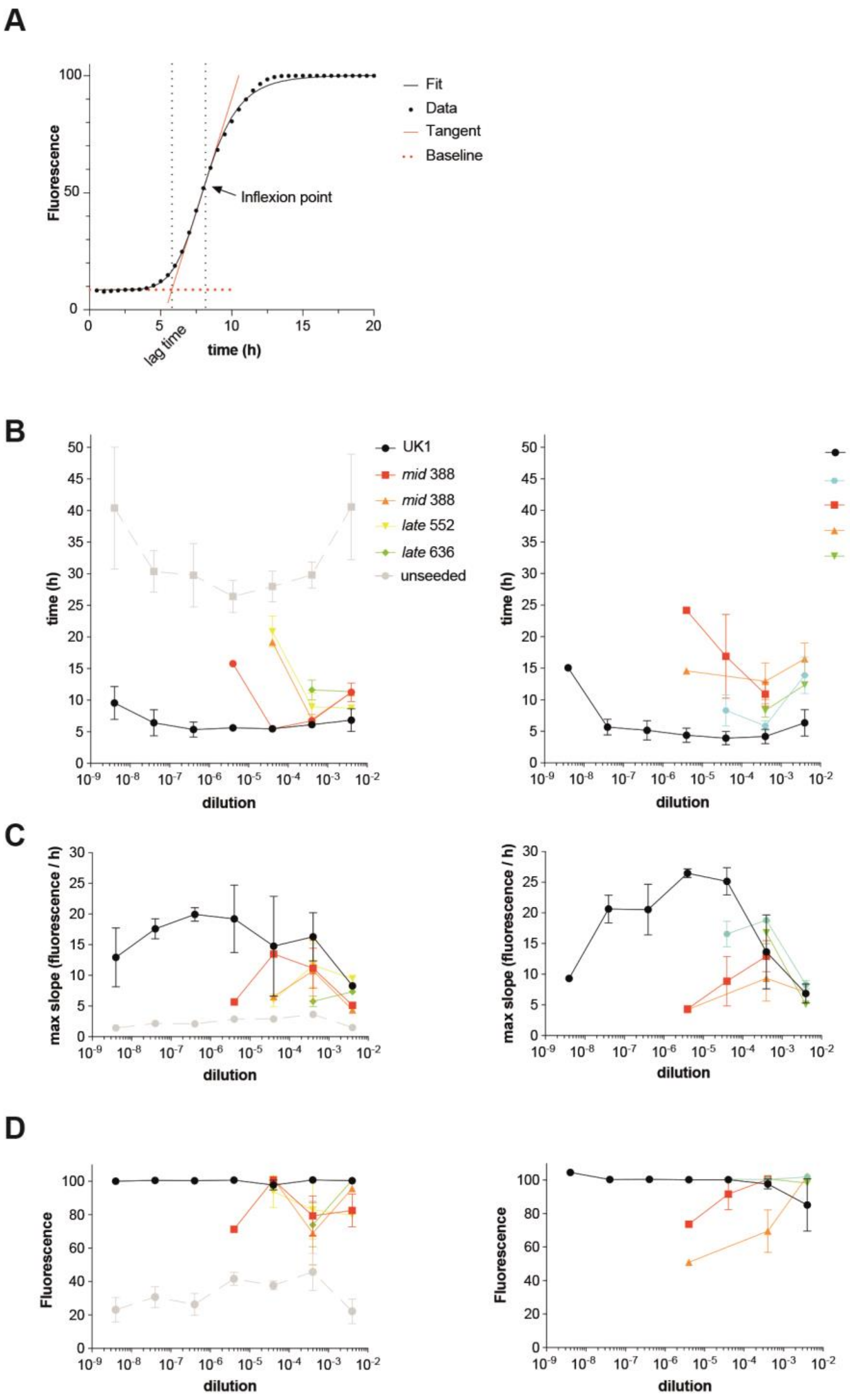

Supplementary Figure 6. Kinetic parameters of RT-QuIC reactions induced by MM1-sCJD prion seeds from PrP$^{0/0}$ mice or human PrP mice

(**A**) Scheme summarizing the kinetic parameters analyzed. Lag phase (**B**), slope at the inflexion point (**C**) and final fluorescence intensity values (**D**) as a function of UK1 (left panel) and Fr2

(right panel) prion concentration in *mid* and *late* brains from $PrP^{0/0}$ mice (n=5) as compared to brains from human PrP tg650 mice at the disease terminal stage (n=3). The days post-inoculation are indicated for *mid* and *late* $PrP^{0/0}$ brains. Each brain sample was serially diluted down to the 10^{-8} dilution. Each trace is the mean ± sd from three to five replicate wells. As control, unseeded reactions are shown (n=5 replicate wells).

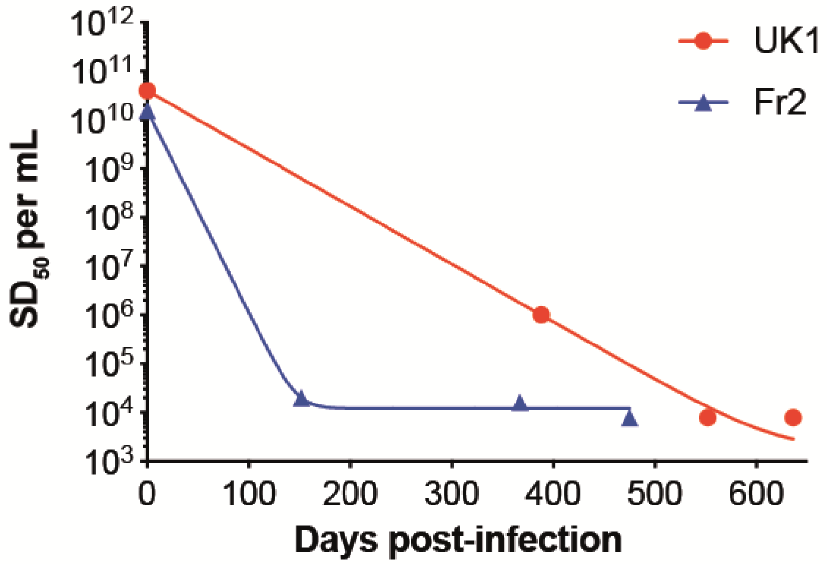

Supplementary Figure 7. PrP^{Sc} half-life in MM1-sCJD inoculated PrP$^{0/0}$ mice, as based on SD$_{50}$ values

Decrease in SD$_{50}$ values per mL of 10% (w/v) brain over time in PrP$^{0/0}$ mice inoculated with UK1 and Fr2 MM1-sCJD prions. SD$_{50}$ values were obtained by RT-QuIC.

References

1. Jaumain E, Quadrio I, Herzog L, et al. Absence of Evidence for a Causal Link between Bovine Spongiform Encephalopathy Strain Variant L-BSE and Known Forms of Sporadic Creutzfeldt-Jakob Disease in Human PrP Transgenic Mice. *J Virol*. Dec 1 2016;90(23):10867-10874. doi:10.1128/JVI.01383-16

2. Beringue V, Vilotte JL, Laude H. Prion agent diversity and species barrier. *Vet Res*. Jul-Aug 2008;39(4):47. doi:10.1051/vetres:2008024
v08241 [pii]

3. Paxinos G, Franklin KBJ. *The mouse brain in stereotaxic coordinates*. Second edition ed. Academic Press; 1997.

4. Beringue V, Le Dur A, Tixador P, et al. Prominent and persistent extraneural infection in human PrP transgenic mice infected with variant CJD. *PLoS One*. 2008;3(1):e1419. doi:10.1371/journal.pone.0001419

5. Cassard H, Torres JM, Lacroux C, et al. Evidence for zoonotic potential of ovine scrapie prions. *Nat Commun*. Dec 16 2014;5:5821. doi:10.1038/ncomms6821
